# Whole-genome sequencing and analysis of the Malaysian cynomolgus macaque (*Macaca fascicularis*) genome

**DOI:** 10.1186/gb-2012-13-7-r58

**Published:** 2012-07-02

**Authors:** Atsunori Higashino, Ryuichi Sakate, Yosuke Kameoka, Ichiro Takahashi, Makoto Hirata, Reiko Tanuma, Tohru Masui, Yasuhiro Yasutomi, Naoki Osada

**Affiliations:** 1 Laboratory of Rare Disease Biospecimen, Department of Disease Bioresources Research, National Institute of Biomedical Innovation, 7-6-8 Saito-asagi, Ibaraki, Osaka 567-0085, Japan; 2 Center for Human Evolution Modeling Research, Primate Research Institute, Kyoto University, Inuyama, Aichi 484-8506, Japan; 3 Tsukuba Primate Research Center, National Institute of Biomedical Innovation, 1-1 Hachimandai, Tsukuba, Ibaraki 305-0843, Japan; 4 Division of Evolutionary Genetics, Department of Population Genetics, National Institute of Genetics, 1111 Yata, Mishima, Shizuoka 411-8540, Japan; 5 Department of Genetics, The Graduate University for Advanced Studies (SOKENDAI), 1111 Yata, Mishima, Shizuoka 411-8540, Japan

## Abstract

**Background:**

The genetic background of the cynomolgus macaque (*Macaca fascicularis*) is made complex by the high genetic diversity, population structure, and gene introgression from the closely related rhesus macaque (*Macaca mulatta*). Herein we report the whole-genome sequence of a Malaysian cynomolgus macaque male with more than 40-fold coverage, which was determined using a resequencing method based on the Indian rhesus macaque genome.

**Results:**

We identified approximately 9.7 million single nucleotide variants (SNVs) between the Malaysian cynomolgus and the Indian rhesus macaque genomes. Compared with humans, a smaller nonsynonymous/synonymous SNV ratio in the cynomolgus macaque suggests more effective removal of slightly deleterious mutations. Comparison of two cynomolgus (Malaysian and Vietnamese) and two rhesus (Indian and Chinese) macaque genomes, including previously published macaque genomes, suggests that Indochinese cynomolgus macaques have been more affected by gene introgression from rhesus macaques. We further identified 60 nonsynonymous SNVs that completely differentiated the cynomolgus and rhesus macaque genomes, and that could be important candidate variants for determining species-specific responses to drugs and pathogens. The demographic inference using the genome sequence data revealed that Malaysian cynomolgus macaques have experienced at least three population bottlenecks.

**Conclusions:**

This list of whole-genome SNVs will be useful for many future applications, such as an array-based genotyping system for macaque individuals. High-quality whole-genome sequencing of the cynomolgus macaque genome may aid studies on finding genetic differences that are responsible for phenotypic diversity in macaques and may help control genetic backgrounds among individuals.

## Background

Cynomolgus macaque (*Macaca fascicularis*) is one of the most commonly used nonhuman primates in biomedical research worldwide [1]. It is also called the crab-eating or long-tailed macaque and belongs to the *fascicularis *group of the genus *Macaca *[2]. A number of pharmaceutical companies use cynomolgus macaques for drug development and, thus, identifying genetic components that contribute to their drug metabolism is a key issue in biomedical genomic research [3,4].

Rhesus macaque (*Macaca mulatta*), whose draft genome sequence was determined by the Sanger sequencing method with a BAC clone assembly [5], is genetically closely related to the cynomolgus macaque. Whereas rhesus macaques occur from India to southern China and in some neighboring areas, cynomolgus macaques can be found throughout Southeast Asia. Vital hybrids of the two macaques have been observed around northern Thailand, supporting their very close genetic relationship [6]. Previous studies have shown that cynomolgus and rhesus macaques share a considerable number of single nucleotide variants (SNVs) [7,8]. Their genetic divergence is estimated to be approximately 0.4% [8,9].

Recently, several genome sequences of macaques have been determined using next-generation sequencing platforms. These include Mauritian and Vietnamese cynomolgus macaques [4,10], two independent Chinese rhesus macaques [10,11] and one Indian rhesus macaque [12]. The two cynomolgus macaque individuals (Mauritian and Vietnamese), however, were derived from two genetically distinct populations that have experienced peculiar demographic histories. Previous studies have suggested that cynomolgus macaques are genetically clustered into Indonesian-Malaysian, Philippine, Indochinese, and Mauritian macaques [8,13]. Mauritian macaques have been known to show extremely low genetic diversity that is associated with their recent colonization [14], whereas Indochinese macaques have experienced a considerable amount of gene flow with rhesus macaques [15,16]. Therefore, the whole-genome sequencing of Indonesian-Malaysian cynomolgus macaques, which show the highest genetic diversity and, according to the fossil evidence, originate from a putative ancestral population [17], would provide significant insight into the genetic differentiation of cynomolgus and rhesus macaques at the species level.

Recent advances in DNA sequencing technologies have enabled rapid and economical determination of whole-genome sequences of organisms. Although *de novo *assemblies of large and complicated genomes, such as mammalian genomes, remain difficult, whole-genome resequencing has become a powerful method for identifying genetic variation within a biological species. Human genome variation is of particular interest for medical and evolutionary studies, and a dozen human genome sequences have thus far been determined using resequencing methods [18-24]. Whole-genome resequencing is not only efficient for identifying variations within a species, but also applicable to closely related species. Because the current methods of mapping short DNA sequence reads have been developed to amend relatively high sequencing errors in massively parallel sequencing, they are also expected to be useful for small sequence divergence. Thus, the strategy of resequencing species that are closely related to model organisms of known genome sequence may be an efficient and important method for detecting genomic diversity.

In this study, we determined and analyzed the Malaysian cynomolgus macaque genome sequence using the massively parallel sequencer SOLiD 3 Plus System (Life Technologies). The sequenced reads were mapped to the Indian rhesus macaque (reference) genome sequence with more than 40-fold coverage. A total of approximately 9.7 million SNVs and 1 million small (< 12 bp) indels and 60,000 large indels (44 to 732 bp) were identified. The identified SNVs were compared with SNVs previously determined for other cynomolgus and rhesus macaque genomes. These SNVs have been deposited in the cynomolgus macaque genome resources database (QFbase [25]). High-quality resequencing of the cynomolgus macaque genome will facilitate further studies directed towards dissecting genetic differences that are responsible for phenotypic divergence among macaque species.

## Results

### Sequencing and mapping

**Figure 1:**
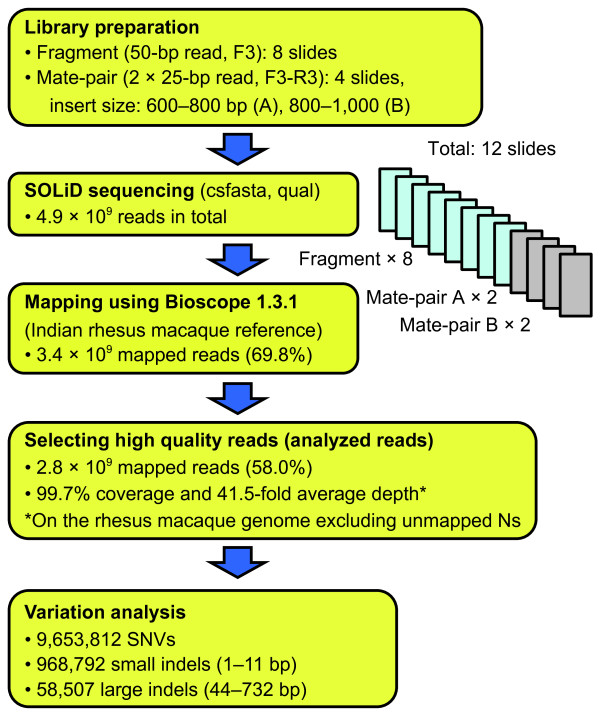
**Overview of the Malaysian cynomolgus macaque genome sequencing and analysis**. Fragment runs of eight slides and mate-pair runs of four slides (insert size: two slides for 600 to 800 bp and two slides for 800 to 1,000 bp) were performed on the SOLiD 3 Plus System. In total, 4.9 × 10^9 ^sequence reads were generated and mapped on the reference genome. After the high-quality reads were selected, single nucleotide variants (SNVs) and indel analyses were conducted.

Blood samples from a 25-year-old male Malaysian cynomolgus macaque were used for genome resequencing. Figure 1 outlines the procedure of the cynomolgus macaque genome resequencing. We performed eight cycles of fragment library sequencing (50 bp) and four cycles of mate-pair library sequencing (2 × 25 bp) using the SOLiD 3 Plus System. The mate-pair libraries of two different insert sizes (600 to 800 bp and 800 to 1,000 bp) were constructed and analyzed. Table 1 summarizes the results of genome sequencing and mapping. A total of 2.6 × 10^9 ^reads of fragment sequence and 2.2 × 10^9 ^reads of mate-pair sequence data were obtained. The mapping program implemented in BioScope software v1.3.1 (Life Technologies) was used for mapping the reads. A total of 3.4 × 10^9 ^reads (69.8%) were successfully mapped on the Golden Path genome assembly, which was derived from an Indian rhesus macaque (mmu_120505). Finally, analyzed reads totaled 1.1 × 10^11 ^bp, and the average coverage depth was 41.5-fold. All chromosomes exceeded 37-fold (Figure S1 in Additional file 1). The analyzed reads covered 99.7% of the reference genome (unmapped Ns were excluded), and 95.8% of the reference genome was covered by at least 10 reads (Figure S2 in Additional file 1). In order to examine whether our mapping statistics depended on the genome assembly, we also mapped our reads to the recently determined Vietnamese cynomolgus macaque genome, which was constructed by *de novo *assembly of short reads [10]. As a result, a similar mapping rate level (67.2%) and genome coverage (42.5-fold) were obtained (Table S1 in Additional file 1). We primarily focus on the results obtained using the Golden Path genome assembly throughout the rest of the paper because the reference genome had more detailed genome annotations, and the results are comparable with those of other studies. Hereafter, we refer to the Golden Path genome assembly as the "reference" genome.

**Table 1:** Summary of SOLiD libraries and sequence reads

Library	Read length (bp)	Insert size (bp)	Runs	Reads	Mapped reads	Analyzed reads^a^	Coverage depth of analyzed reads
Fragment	50	-	8	2,648,128,521	1,976,720,560 (74.7%)	1,974,496,337 (74.6%)	33.4
Mate-pair A	25 (×2)	600-800	2	906,783,481	621,175,871 (68.5%)	355,589,008 (39.2%)^b^	3.4
Mate-pair B	25 (×2)	800-1,000	2	1,335,583,547	814,866,634 (61.0%)	508,168,736 (38.0%)^b^	4.8
Total	-	-	12	4,890,495,549	3,412,763,065 (69.8%)	2,838,254,081 (58.0%)	41.5

^a^Reads mapped on chrM and chrUr were removed. ^b^'PCR or optical duplicates' (defined by Bioscope; mapped more than 100 loci) were removed, and properly paired reads were selected; each read of a pair was mapped on the same chromosome in a proper direction at a proper distance from each other.

### Single nucleotide variant detection

**Figure 2:**
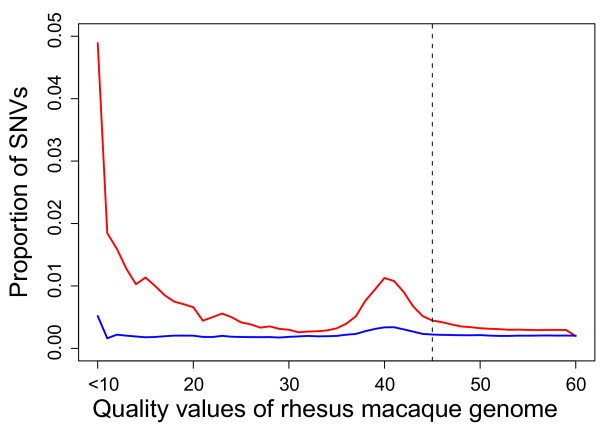
**SNV discovery rate and rhesus macaque genome quality**. The red and blue lines represent the rates of homozygous and heterozygous SNVs, respectively, with given rhesus macaque genome sequence quality values (QVs). SNVs at sites having QV < 45 (left of the dashed line) were filtered out.

SNVs were called with SAMtools [26] using the mapped reads on the reference genome. SNVs at low (< 5) coverage sites and with low call quality values (QV < 40) were excluded. Because the reference genome sequence has not yet been finalized, we examined the relationship between the quality of the reference genome assembly and the SNV discovery rate. We expected that homozygous SNVs in low-quality genomic regions were possible errors in the reference genome sequence and that heterozygous SNVs were robust in genome quality. As shown in Figure 2, we plotted the proportions of homozygous and heterozygous SNVs against the reference genome QVs. Although the heterozygous SNV discovery rate was nearly constant across genome quality, homozygous SNV rates in low-quality regions were relatively high, suggesting that those SNVs were probably due to errors in the genome sequence and should be filtered out. In addition, we observed a slight peak in homozygous SNV rates at QV around 40. This pattern was also observed when we removed SNVs within repeat regions (data not shown) and may have been due to unknown problems in the assembly process of the reference genome sequence. Based on this observation, we decided to filter out SNVs at sites having QVs < 45 in the reference genome sequence. This filtering did not significantly sacrifice our SNV detection power, because > 94% of the reference rhesus macaque genome had QV = 60.

Using the above criteria, we identified 4,880,874 heterozygous and 4,527,169 homozygous SNVs on autosomes. The number of estimated SNVs is summarized in Table 2. Note that the numbers in this table are underestimates because SNVs ambiguously assigned as either homozygous or heterozygous were not included (see Materials and methods). In autosomal non-coding regions, 42,930 untranslated exonic (5'/3' UTR), 2,878,903 intronic, and 6,422,898 intergenic SNVs were identified. Among them, 3,707,670 SNVs were mapped to repeat regions. The nucleotide change pattern of the SNVs is shown in Table S2 in Additional file 1. The transition-to-transversion ratio was 2.39, which is close to the estimated value in humans [27]. SNV densities on chromosomes are summarized in Figure S3 in Additional file 1. Using the same SNV-detecting criteria, we identified about 8.5 million SNVs by mapping Malaysian cynomolgus macaque reads on the Vietnamese cynomolgus macaque genome sequences.

**Table 2:** Number of single nucleotide variants

Chromosome	Heterozygous SNVs	Homozygous SNVs	A^a^	S^b^	UTR^c^	Intronic	Intergenic
Autosomes	4,880,874	4,527,169	25,079	38,233	42,930	2,878,903	6,422,898
X chromosomes	-^d^	245,769	444	701	986	50,877	192,761
Total	4,880,874	4,772,938	25,523	38,934	43,916	2,928,970	6,615,659

^a^Number of nonsynonymous SNVs. ^b^Number of synonymous SNVs. ^c^Number of SNVs in untranslated regions. ^d^Only homozygous SNVs were considered on the X chromosome.

Among 18,912 annotated autosomal protein-coding genes, 14,560 carried at least one coding SNV, consisting of 25,079 nonsynonymous and 38,233 synonymous SNVs. We found that 9,753 autosomal genes contained at least one heterozygous or homozygous amino acid variation in the Malaysian cynomolgus macaque genome, compared with the reference rhesus macaque genome. In addition, 108 and 200 autosomal genes harbored nonsense mutations that were homozygous and heterozygous, respectively. We also estimated the number of SNVs on the X; chromosome. Only homozygous SNVs on the X chromosome were counted. In total, we identified 245,769 SNVs on the X; chromosome, including 1,145 coding (444 nonsynonymous and 701 synonymous SNVs in 662 protein-coding genes), 986 UTR, 50,877 intronic, and 192,761 intergenic homozygous SNVs (Table 2).

### Comparisons with previously determined macaque genomes

The newly identified whole-genome SNVs between Malaysian cynomolgus and Indian (reference) rhesus macaques were compared with previously identified SNVs. We downloaded short-read sequences of Vietnamese cynomolgus and Chinese rhesus macaques that had comparable coverage depth to ours (> 40-fold) and mapped on the reference genome [10]. Using the same SNV-detecting pipeline, we identified 13,244,140 and 10,662,418 SNVs in the Vietnamese cynomolgus and Chinese rhesus macaque genomes, respectively. The Malaysian cynomolgus macaque shared 5,181,509 SNVs with the Vietnamese cynomolgus macaque, either homozygous or heterozygous, showing that > 50% of our SNVs were shared between the two cynomolgus macaque individuals. Merging the two cynomolgus macaque genomes yielded 17,716,443 SNVs in cynomolgus macaques. Furthermore, we found that 2,519,988 SNVs were restricted to the Malaysian cynomolgus macaque, and 1,368,528 SNVs were completely differentiated between the two cynomolgus and two rhesus macaque genomes. Because sequencing platforms and coverage depth differed among the studies, we could not directly compare the number of inferred SNVs. We therefore compared the fraction of heterozygous SNVs shared between two genomes. About 8% of Malaysian and 11% of Vietnamese heterozygous SNVs were also heterozygous SNVs in the Chinese rhesus macaque, supporting the contention that Indochinese cynomolgus macaques have been more vulnerable to gene introgression from rhesus macaques than Indonesian-Malaysian macaques.

We next searched for immune- and drug-response genes that carried nonsynonymous SNVs in the Malaysian cynomolgus macaque, because these genes are of particular interest in biomedical research. In total, 72 and 42 autosomal genes, of which the human orthologs had been annotated as immune-response (GO: 0006955) and drug-response (GO: 0042493) genes, respectively, had at least one homozygous amino acid change in the Malaysian cynomolgus macaque genome. We further checked whether these homozygous SNVs were likely to be differentiated between the two macaque species. A handful of genes, 29 immune- and 18 drug-response genes, carried completely segregating nonsynonymous SNVs between cynomolgus and rhesus macaques, for a total of 60 nonsynonymous SNVs (Table S3 in Additional file 1).

### Population genetic inferences from resequenced data

In contrast to previous resequencing studies, the reference genome and the resequenced genome in this study were from highly differentiated but not completely isolated populations. The average genetic diversity in cynomolgus macaques (nucleotide diversity) corresponded to the fraction of heterozygous SNVs (differences between two sequenced chromosomes) if there was no consanguinity effect, whereas the average genetic divergence between species (Nei's *d_xy_*) [28] corresponded to the fraction of homozygous SNVs plus one-half of the heterozygous SNVs.

In order to infer the strength of natural selection within and between macaque species, we estimated the ratio of nonsynonymous to synonymous SNVs. The ratio of nonsynonymous to synonymous heterozygous SNVs within cynomolgus macaques was 0.68. In order to compare the ratios in macaques and humans, a diploid human genome sequence determined by a short-read sequencer with similar read depth (African genome, NA19839) was retrieved from the public database. The human SNVs were determined using the same SNV-detecting pipeline described above. The ratio of nonsynonymous to synonymous heterozygous SNVs in the African human genome was 0.89, significantly higher than the ratio in the macaque (*P *< 10^-15^, chi-square test). This pattern agrees well with the nearly neutral theory, in which slightly deleterious mutations tend to be segregated within small populations [29], because these macaques have four to five times larger effective population sizes than extant humans. In addition, the ratio within cynomolgus macaques (0.68) was slightly but statistically and significantly higher than that between cynomolgus and rhesus macaques (0.65; *P *= 0.002, chi-square test). If most of the nonsynonymous SNVs between cynomolgus and rhesus macaques were due to diversifying selection between species, the ratio of nonsynonymous to synonymous SNVs between species should be higher than that within species. This pattern also could be explained by the nearly neutral theory, wherein slightly deleterious mutations are short-lived and cannot contribute to species differentiation.

### Small indels detected by sequence mapping

**Figure 3:**
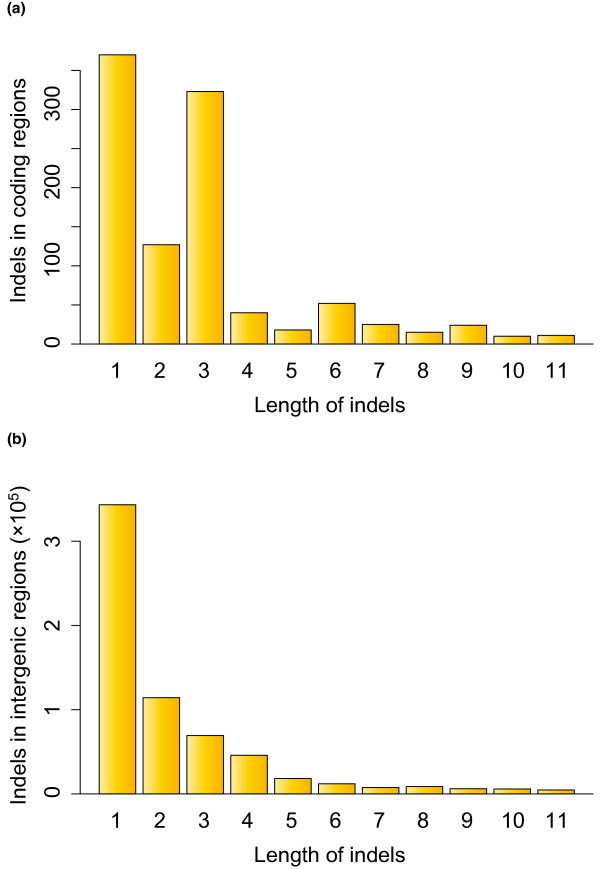
**Distribution of small-indel lengths identified in the Malaysian cynomolgus macaque genome**. **(a) **Indels in coding regions. **(b) **Indels in intergenic regions. The indels were identified using the information from high-quality reads.

Using the mapping information of sequence reads, we also estimated the number of small indels (< 12 bp) in the Malaysian cynomolgus macaque genome. Interestingly, we observed a slight increase in small indels around QV = 40 of the reference genome sequence (Figure S4 in Additional file 1). We therefore filtered out small indels at sites with QV < 45 in the reference sequence. In total, we identified 365,581 insertions and 587,456 deletions on autosomes and 7,023 insertions and 8,732 deletions on the X chromosome. Only homozygous indels were counted on the X chromosome. Out of 372,604 small insertions and 596,188 small deletions in total, 154,649 (42%) and 250,398 (42%) were assigned to repeat regions, respectively. Among 1,139 indels within autosomal protein-coding regions, 705 were frameshifting and 434 were non-frameshifting (3x-bp-length) indels. The proportion of 3x-bp-length indels (38%) was significantly higher than the value expected from intergenic indels (14%; *P *< 10^-15^, chi-square test), suggesting purifying selection on frameshifting indels in coding regions. The distribution of small indel lengths is shown in Figure 3.

### Large indels detected by mate-pair distance

An early chromosome-banding study suggested a paracentric chromosomal inversion in the long arm of chromosome 5 between cynomolgus and rhesus macaques [30]. In order to examine the occurrence of inversion at the chromosome-banding level (> 1 Mb), we surveyed mate-pair sequence reads that were not properly aligned on chromosome 5. The number of mate-pair reads showing the signature of inversion was counted within 500-bp-length windows with 250-bp sliding steps. In total, 28 windows that contained ≥ 50 incongruent reads were found. However, all of the windows included alpha satellite repeats and none showed evidence of the large inversion.

We further analyzed the pattern of large insertions and deletions using the information from the mate-pair libraries of different insert sizes (mate-pair library A, 600 to 800 bp; library B, 800 to 1,000 bp). A total of 29,009 and 50,945 indels were identified using libraries A and B, respectively. Merging these indels yielded 8,301 insertions and 50,206 deletions; the insertion and deletion size ranges were 77 to 732 bp and 44 to 601 bp, respectively. Although the reference genome assembly has consecutive indices for each chromosome, the assembled genome sequences were built from scaffolds and contigs connected with assembly gaps (stretches of Ns). Among the 50,206 deletions, 45,821 and 22,774 encompassed repeat sequences and ambiguous sequences, respectively. Similarly, among the 8,301 insertions, 7,886 and 1,729 were within repeat sequences and ambiguous sequences, respectively. The distributions of insertion and deletion lengths that were not associated with gaps are shown in Figure S5 in Additional file 1.

### Inference of demography

**Figure 4:**
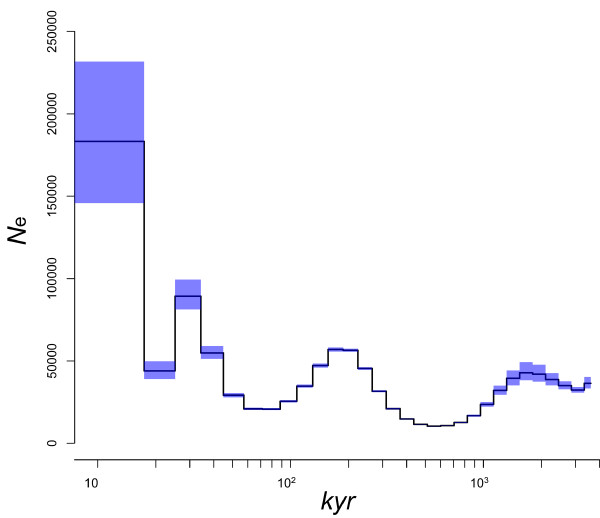
**Estimation of ancestral population size of cynomolgus macaques with PSMC software**. The *x *and *y *axes show population size and the time (thousand years (kyr)) from present, respectively. The blue rectangles represent 95% confidence interval with bootstrap resampling.

Recently, Li and Durbin [31] developed a novel method for inferring the demography of species from single diploid genome data. The demography is inferred from a distribution of coalescence time between two haploid genomes. We applied this method to our Malaysian cynomolgus genome data, with a generation time of 6 years and a mutation rate per generation of 2.5 × 10^-8^. Figure 4 shows the inferred demography of the cynomolgus macaque with bootstrap 95% confidence interval. Although the scaling parameters affect the estimation of time and population size scales, the result showed at least three population bottlenecks in the past. In agreement with the previous estimates, the cynomolgus macaque population size expanded more than several fold during a million-year period [8,10,32].

### Database resource

**Figure 5:**
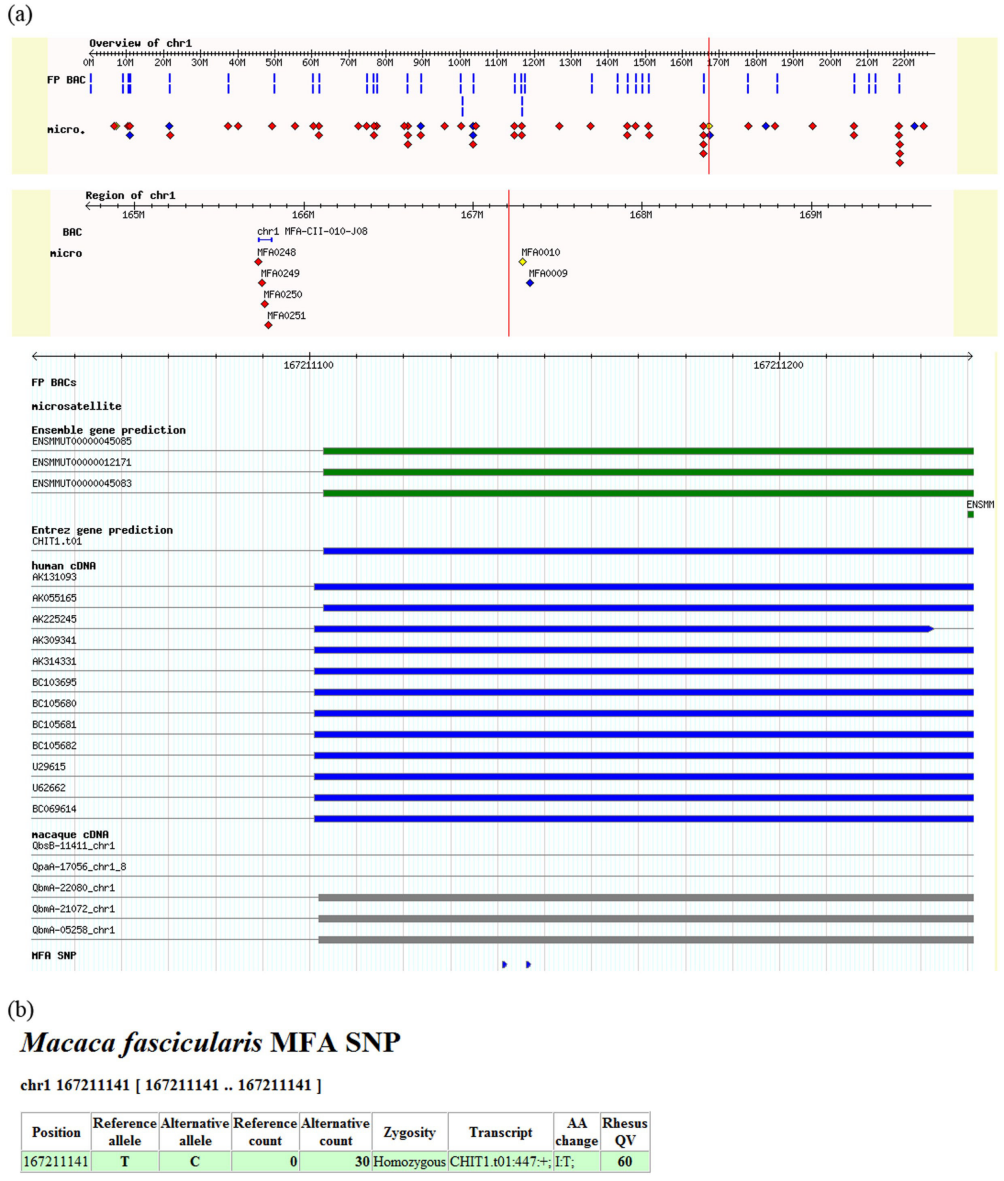
**Screenshots of the macaque genome database**. **(a) **cDNA clones, BAC clones, microsatellite markers, gene predictions, and SNVs on chromosome 1 are shown in the genome browser. **(b) **Detailed information on each SNV is linked from the browser.

The Malaysian cynomolgus macaque genome sequence reads have been deposited to public databases (DDBJ Sequence Read Archive: DRA000430), and identified SNVs have been registered to the *Macaca fascicularis *genome database (QFbase [25]), which was previously built by our research group. The database was constructed based on the reference genome sequence of the Indian rhesus macaque, and the annotation of cynomolgus macaques was implemented, including cDNA sequences, BAC clones, and microsatellite markers [9,33]. An example of a graphical view of SNVs in the browser is shown in Figure 5. Because cynomolgus macaques are frequently used in animal experiments, these resources will be valuable for researchers who are not familiar with large-scale data manipulation.

## Discussion

Controlling the genetic background of experimental animals is a key issue for the efficiency and reliability of preclinical trials in biomedical research. Previous studies have shown that macaques, which are the most popular primates for biomedical research, harbor much higher genetic diversity than humans, even if they are collected from a limited area [8,15,32]. Thus, high-quality whole-genome sequences of cynomolgus macaques are necessary for future biomedical studies in order to control and quantify differences in genetic backgrounds. In addition, many morphological and physiological differences have been reported between the macaque species, including behaviors, tail lengths, body sizes, and susceptibility to pathogens and drugs [34,35]. Determining genetic differences between cynomolgus and rhesus macaques that contribute to phenotypic differences between them is an important subject for both biomedical and evolutionary research.

In this study, we have identified about 9.7 million SNVs between Malaysian cynomolgus and Indian rhesus macaques and 8.5 million SNVs between Malaysian and Vietnamese cynomolgus macaques. The total number of SNVs is much higher than that estimated in human genome resequencing studies (approximately 3 million). Although we cannot directly compare the number of SNVs determined with different platforms and different inference methods, the high level of genetic diversity within macaque species is in agreement with previous multi-locus sequencing studies using the Sanger method [8,32] and with the whole-genome sequencing study using a different platform with a similar level of genome coverage [10]. Despite the high level of genetic diversity within and between macaque species, the number of SNVs potentially responsible for species delimitation may be limited, partly owing to frequent gene flow between Indochinese cynomolgus and Chinese rhesus macaques. Only about 10% of SNVs were completely segregated between the two cynomolgus and two rhesus macaque genomes, which were further narrowed down to 60 nonsynonymous SNVs in drug- and immune-related genes.

The number of nonsynonymous SNVs was also higher in macaques than in humans. Whereas about 10,000 nonsynonymous SNVs were segregated in humans, about 30,000 nonsynonymous SNVs were segregated within and between macaque species. Interestingly, the level of protein diversity relative to background genetic diversity in macaques was significantly smaller than that expected from human data. This difference is probably due to the large effective population size of macaques, which removes slightly deleterious mutations in populations with relatively better efficiency.

Although we found a considerable number of SNVs and indels with high mapping support, we should be careful of some aspects of the quality of the reference genome assembly. In the large indel analysis using the mate-pair libraries, ≥ 90% of large indels included repeat sequences in the genome, indicating that these are potential repeat regions for genome-size change. Unfortunately, because the data we obtained using the SOLiD platform are not suitable for *de novo *assembly of a whole-genome sequence, we cannot conclude whether or not these hotspots are due to artifacts stemming from the reference genome quality. *De novo *assembly of a whole mammalian genome sequence remains costly, but studies using multiple genomes with *de novo *assembly would elucidate the complex pattern of genome-size changes [10].

The demography of the Malaysian cynomolgus macaque reveals the complex history of macaque genomes. As geological and fossil evidence has suggested, ancestors of the cynomolgus macaque lived in Sundaland, which was created by sea-level lowering during the glacial period [17,36]. The most recent population bottleneck around 20,000 years ago may correspond to the last glacial maximum, when average temperatures were 2 to 6°C lower than the present temperatures. The change in population size is possibly associated with admixture with the rhesus macaque, since their habitats were largely connected by the formation of Sundaland. However, it should be noted that the time estimation largely depends on the generation time parameter of macaques. If we adopt a longer generation time parameter - for example, 10 to 12 years as the median age of females giving offspring - the most recent bottleneck event would shift earlier, 33,000 to 40,000 years ago.

## Conclusions

We identified 9.7 million high-quality SNVs between the Malaysian cynomolgus and the reference (Indian rhesus) macaque genomes. The list of whole-genome SNVs will be useful for many future applications, such as an array-based genotyping system of macaque individuals. In contrast to humans, the genetic variation of experimental animals, especially of monkeys, is largely unexplored. The whole-genome sequence of a Malaysian cynomolgus macaque has unveiled hidden genetic variations among these widely used experimental animals and will benefit future evolutionary and biomedical studies.

## Materials and methods

### Animal and blood sampling

Whole blood cells for genomic DNA were obtained from a 25-year-old male cynomolgus macaque (Malaysian), housed at the Tsukuba Primate Research Center (TPRC), National Institute of Biomedical Innovation (NIBIO), Tsukuba, Ibaraki, Japan, in accordance with the TPRC guidelines. The sampled macaque was an F1 progeny of unrelated wild individuals captured in the south of Kuala Lumpur. These macaques were cared for and handled according to the guidelines established by the Institutional Animal Care and Use Committee of NIBIO and the standard operating procedures for macaques at the TPRC. Blood collection was conducted at the TPRC in accordance with the guidelines of the Laboratory Biosafety Manual, World Health Organization. Genomic DNA was isolated from 10 ml of peripheral blood with EDTA using a Qiagen Genomic DNA purification kit (Qiagen K. K., Tokyo, Japan). The isolated DNA samples were kept at -80°C until use.

### Genome sequencing

Genome sequencing was performed using the SOLiD 3 Plus System (Life Technologies, Gaithersburg, MD, USA). Fragment (50 bp) and mate-pair (25 bp × 2) libraries were generated using the macaque genomic DNA. Mate-pair libraries of 600 to 800 bp and 800 to 1,000 bp insert sizes were prepared, and each library was run in two slides. Library preparations and all SOLiD runs were performed as per the standard manufacturer's protocols.

### Mapping sequence data on the Indian rhesus macaque genome

SOLiD sequence data were mapped on the rhesus macaque draft genome sequence (GenBank accession numbers NC_007858 to NC_007878). The assembly QV of the genome was retrieved from the UCSC website [37]. The reads were mapped using the BioScope (Life Technologies) local alignment algorithm with parameters of 25 bp seed length, 2 mismatches in a seed, and mismatch penalty score -2.0 (default threshold). The algorithm finds genomic regions that match to the first 25 bp of each read, allowing at most 2 mismatches, and extending the regions until the score exceeds the threshold. 'PCR and optical duplicates' reads (defined by BioScope; mapped to more than 100 loci, duplicates) and mate-pair reads incongruently mapped on the reference genome (unpaired reads) were filtered out. All mapped sequence reads were deposited to public databases (DNA data bank of Japan (DDBJ) Sequence Read Archive: DRA000430). Chinese rhesus macaque and Vietnamese cynomolgus macaque genome sequences were downloaded from the public database (accession numbers SRA023855 and SRA023856) and aligned to the rhesus macaque genome sequence using the Bowtie 2 program [38] with a local alignment algorithm. A pre-aligned African genome sequence (NA19239) was retrieved from the 1000 Genomes project website [39]. In all resequenced genomes, SNVs were called using SAMtools with a default parameter setting, except for a mismatch tuning parameter (option -C) of 50.

### Indel detection

The detection and calling of small and large indels were performed using the software implemented in BioScope software v1.3.1. Briefly, small indels were identified using sequence reads mapped with alignment gaps, and large indels were identified using incongruent distances between mate-pair reads. The small indel-finding algorithm could detect deletions shorter than 12 bp and insertions shorter than 4 bp. In both analyses, a default setting of parameters was applied.

### Gene annotation

Entrez Gene annotations in the National Center for Biotechnology Information database were used for classifying SNVs into annotations [40]. Genes assigned to multiple genomic loci were excluded from the analysis. Among 27,424 annotated transcripts in the Indian rhesus macaque genome, 944 showed inconsistencies with the draft genome sequence and were removed from further analyses. When we counted the number of variants at a site with overlapping annotations, we assigned an order of priority as follows: coding exon > non-coding exon > intron > intergenic. For example, when a site was annotated as a coding exon of some transcripts and as an intron of the others, the site was classified as a coding exon. In total, 19,574 protein-coding genes, consisting of 26,480 transcripts, were analyzed. Orthologous genes between human and macaque were determined using the annotations of the Ensembl database [41]. Only one-to-one orthologs were used for subsequent analyses.

### Estimation of demographic parameters

We used PSMC (pairwise sequentially Markovian coalescent) software to infer the demographic history of the Malaysian cynomolgus macaque [31]. Briefly, the program estimates the distribution of coalescent time between two haploid genomes, deduced from the rate of heterozygous SNVs across the genome sequence, with ancestral recombination events inferred by the hidden Markov model. The following parameters were used: time interval = 6 + 29*2, generation time = 6, mutation rate per generation = 2.5 × 10^-8^, and the number of iterations = 25. The 95% confidence intervals were estimated using 200 times bootstrap resampling of 5 Mb genome blocks.

## Abbreviations

BAC: bacterial artificial chromosome; QV: quality value; SNV: single nucleotide variant; UTR: untranslated region.

## Competing interests

The authors declare that the research was conducted in the absence of any commercial or financial relationships that could be construed as a potential conflict of interest.

## Authors' contributions

AH, RS, TM, YY and NO contributed to the design of this research. AH, YK, IT, RT and NO performed the experiments. AH, RS, MH and NO contributed to data analysis. AH, RS and NO wrote the manuscript. All authors read and approved the final manuscript.

## Supplementary Material

Additional file 1 **Figures S1 to S5 and Tables S1 to S3**. Figure S1: chromosomal distribution of fold coverage of quality controlled mapped reads (duplicates and unpaired mate-pair reads were filtered out) on the reference rhesus macaque genome are shown. All chromosomes exceed 37-fold. Figure S2: minimum coverage of quality controlled mapped reads (duplicates and unpaired mate-pair reads were filtered out) on the reference rhesus macaque genome is shown. Genomic regions with at least five-fold coverage were used in the SNV analysis. Figure S3: SNV density along each chromosome. The red and blue lines represent the number of heterozygous and homozygous SNVs in 1 Mb windows, respectively. The step size of window sliding was 100 kb. Figure S4: small indel discovery rate and rhesus macaque genome quality. The red and blue lines represent the rate of small deletions and insertions, respectively, with given rhesus macaque genome sequence quality values (QVs). Small indels at sites having QV < 45 in the rhesus macaque genome sequence were filtered out. Figure S5: distribution of large-indel lengths identified in the cynomolgus macaque genome. Indels were identified using the distance information from the mate-pair libraries. Indel regions containing ambiguous genome sequences were excluded. Table S1: summary of SOLiD libraries and sequence reads (mapped to the Vietnamese cynomolgus macaque genome sequence). Table S2: pattern of nucleotide changes. Table S3: immune- and drug-response genes with completely segregating nonsynonymous SNVs between cynomolgus and rhesus macaques. Click here for file
