# Supplementary material for: Whole-genome sequencing and analysis of the Malaysian cynomolgus macaque (Macaca fascicularis) genome
Source: Genome Biol. 2012 Jul 2;13(7):R58. doi: 10.1186/gb-2012-13-7-r58 (PMC3491380; doi:10.1186/gb-2012-13-7-r58)
Supplement: Additional file 1 — Figures S1 to S5 and Tables S1 to S3. Figure S1: chromosomal distribution of fold coverage of quality controlled mapped reads (duplicates and unpaired mate-pair reads were filtered out) on the reference rhesus macaque genome are shown. All chromosomes exceed 37-fold. Figure S2: minimum coverage of quality controlled mapped reads (duplicates and unpaired mate-pair reads were filtered out) on the reference rhesus macaque genome is shown. Genomic regions with at least five-fold coverage were used in the SNV analysis. Figure S3: SNV density along each chromosome. The red and blue lines represent the number of heterozygous and homozygous SNVs in 1 Mb windows, respectively. The step size of window sliding was 100 kb. Figure S4: small indel discovery rate and rhesus macaque genome quality. The red and blue lines represent the rate of small deletions and insertions, respectively, with given rhesus macaque genome sequence quality values (QVs). Small indels at sites having QV < 45 in the rhesus macaque genome sequence were filtered out. Figure S5: distribution of large-indel lengths identified in the cynomolgus macaque genome. Indels were identified using the distance information from the mate-pair libraries. Indel regions containing ambiguous genome sequences were excluded. Table S1: summary of SOLiD libraries and sequence reads (mapped to the Vietnamese cynomolgus macaque genome sequence). Table S2: pattern of nucleotide changes. Table S3: immune- and drug-response genes with completely segregating nonsynonymous SNVs between cynomolgus and rhesus macaques. [file gb-2012-13-7-r58-S1.DOC]

**Additional files**


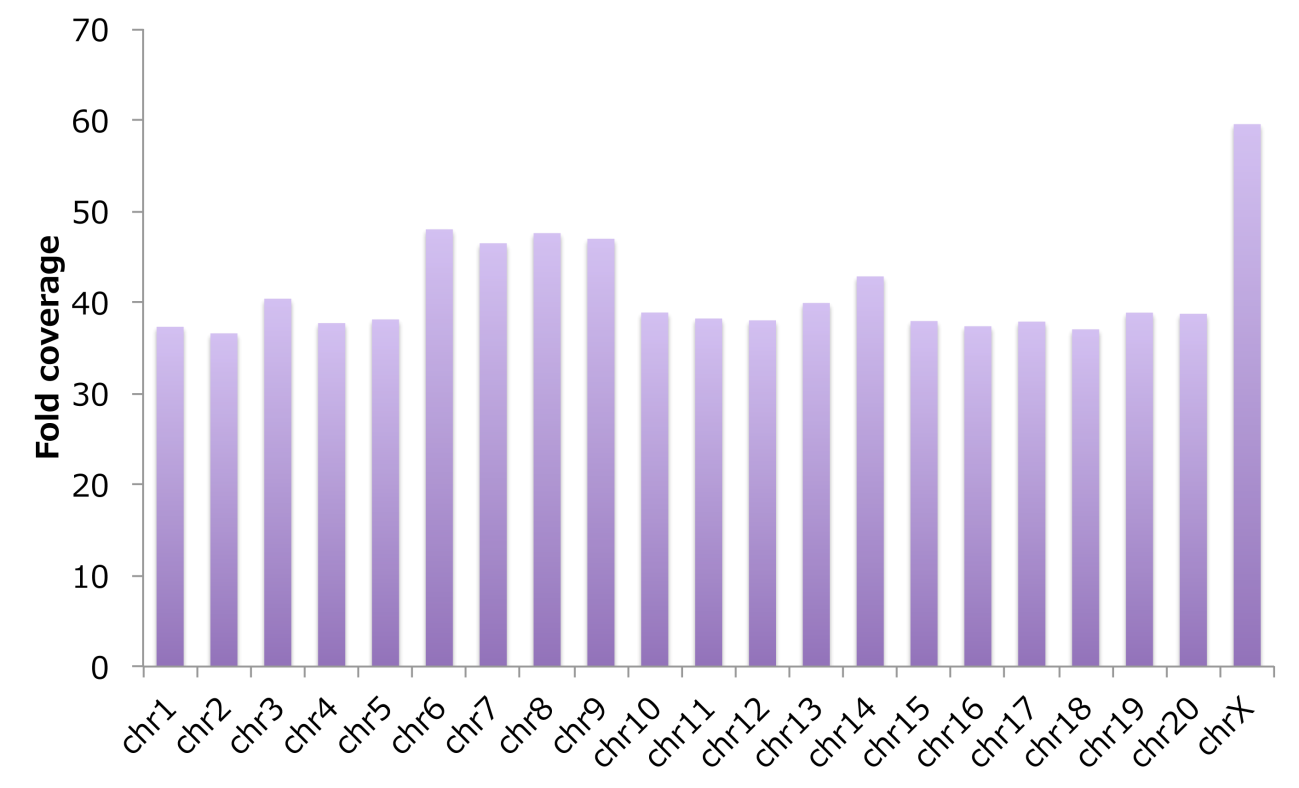


**Supplementary figure 1**

Chromosomal distribution of fold coverage of quality controlled mapped reads (duplicates and unpaired mate-pair reads were filtered out) on the reference rhesus macaque genome are shown. All chromosomes exceed 37-fold.


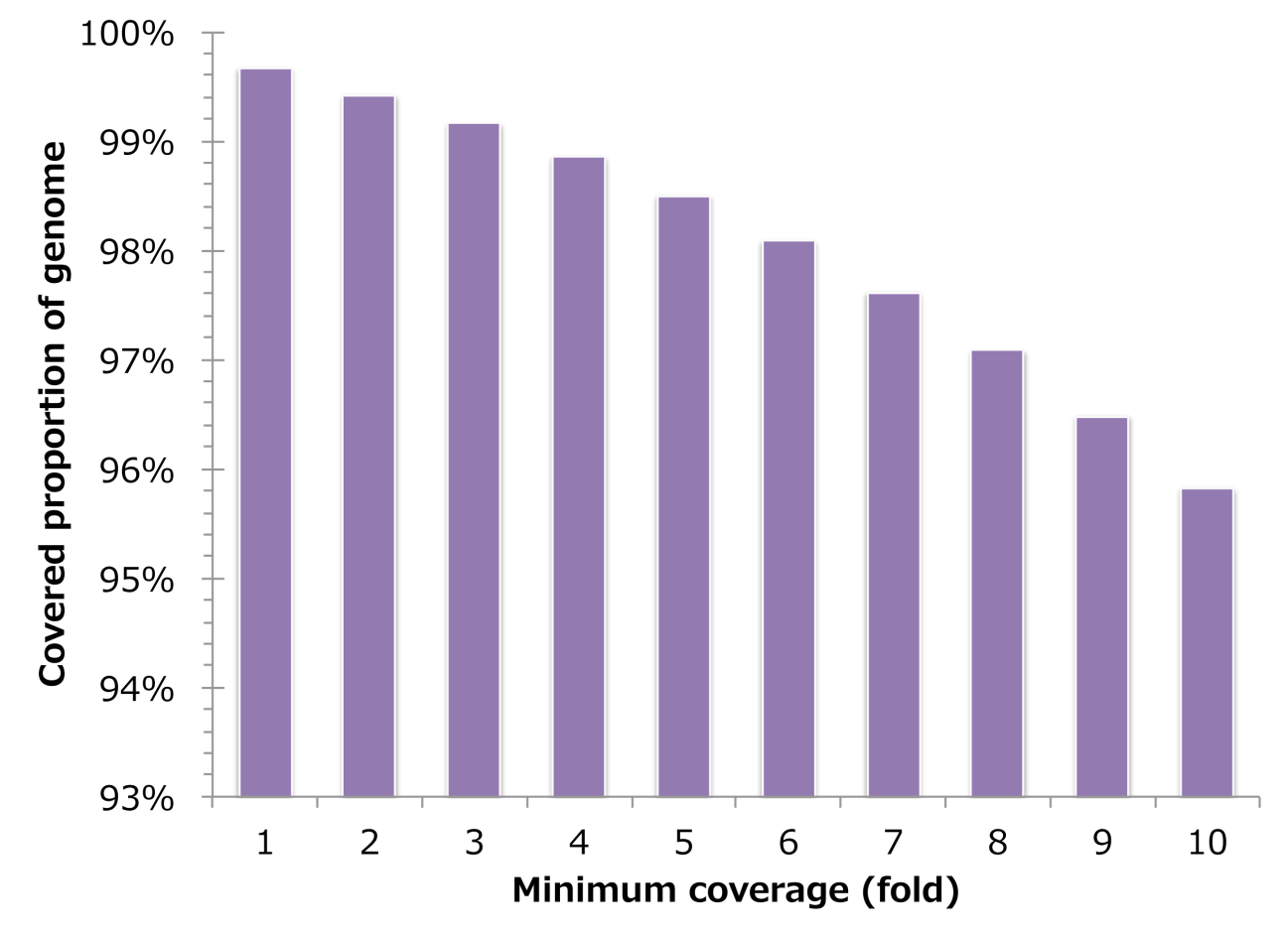


**Supplementary figure 2**

Minimum coverage of quality controlled mapped reads (duplicates and unpaired mate-pair reads were filtered out) on the reference rhesus macaque genome is shown. Genomic regions with at least five-fold coverage were used in the SNV analysis.


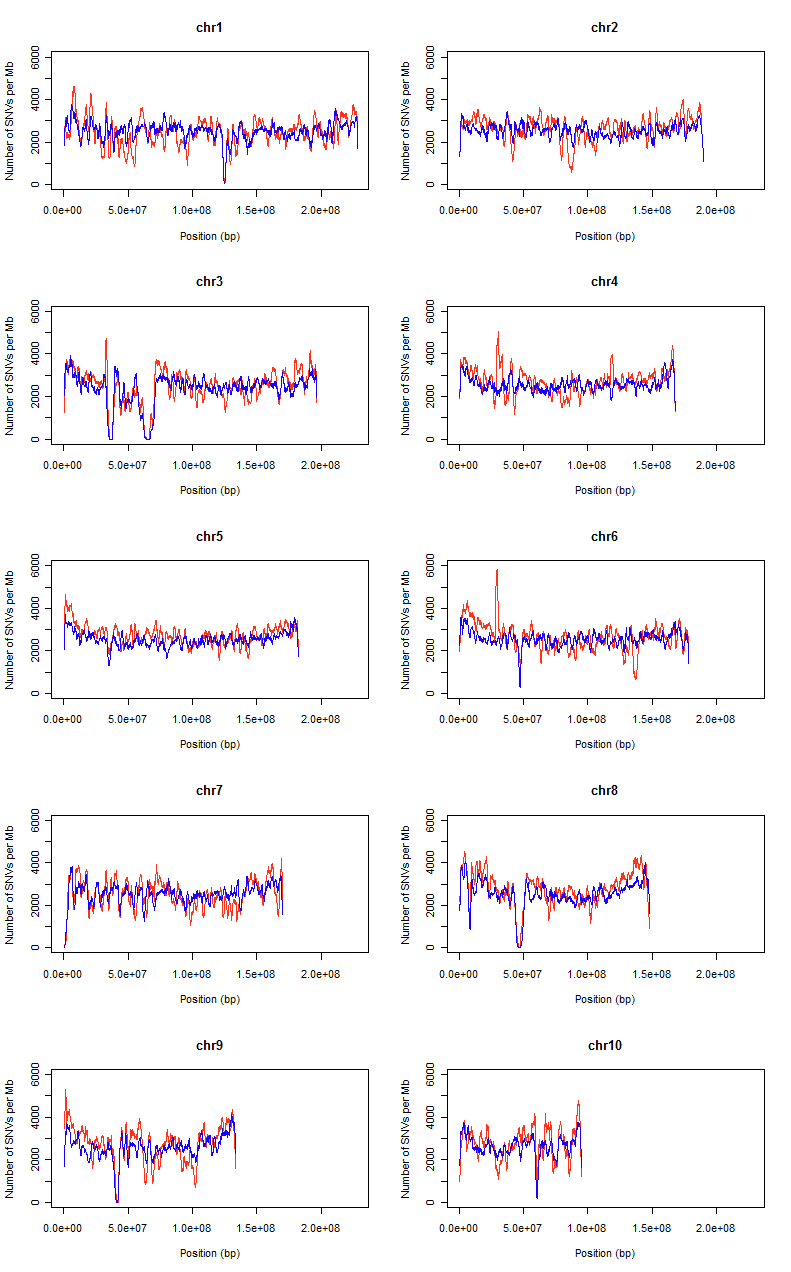


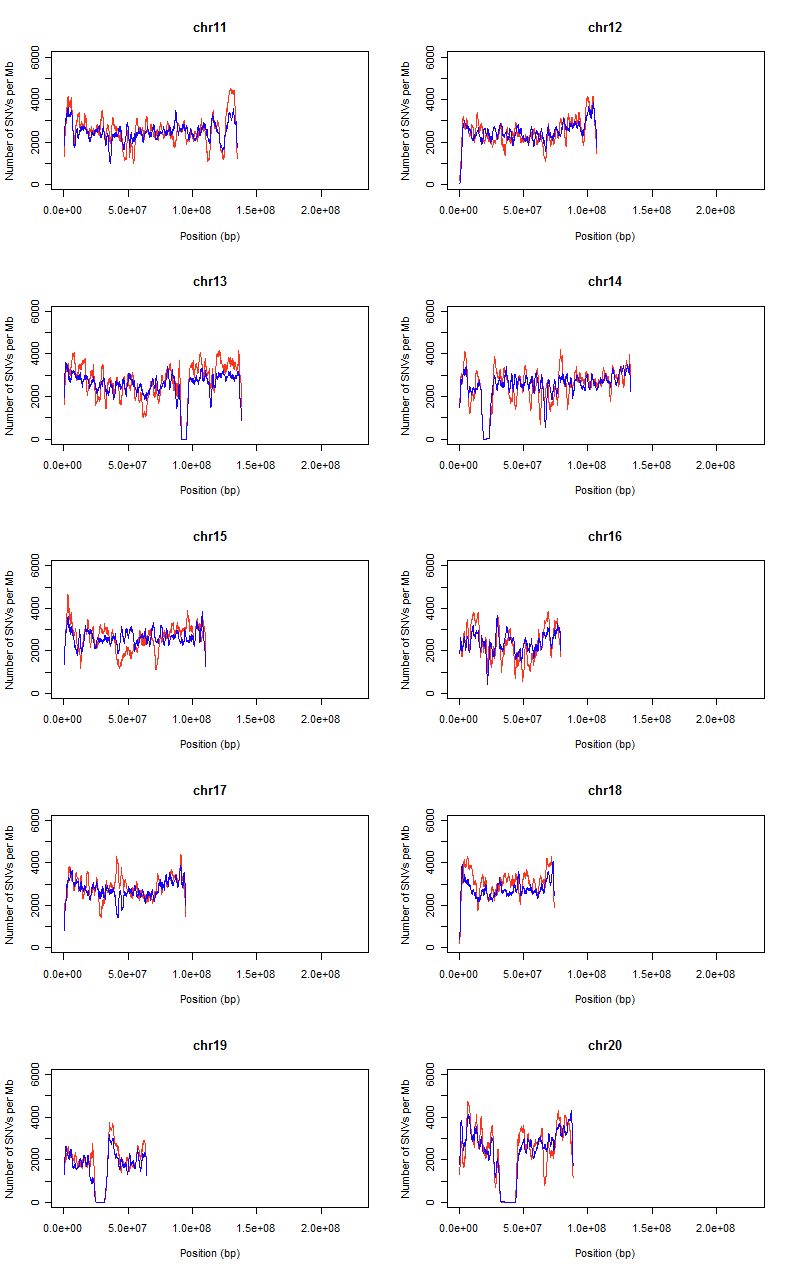


**Supplementary figure 3**

SNV density along each chromosome. The red and blue lines represent the number of heterozygous and homozygous SNVs in 1 Mb windows, respectively. The step size of window sliding was 100 kb.


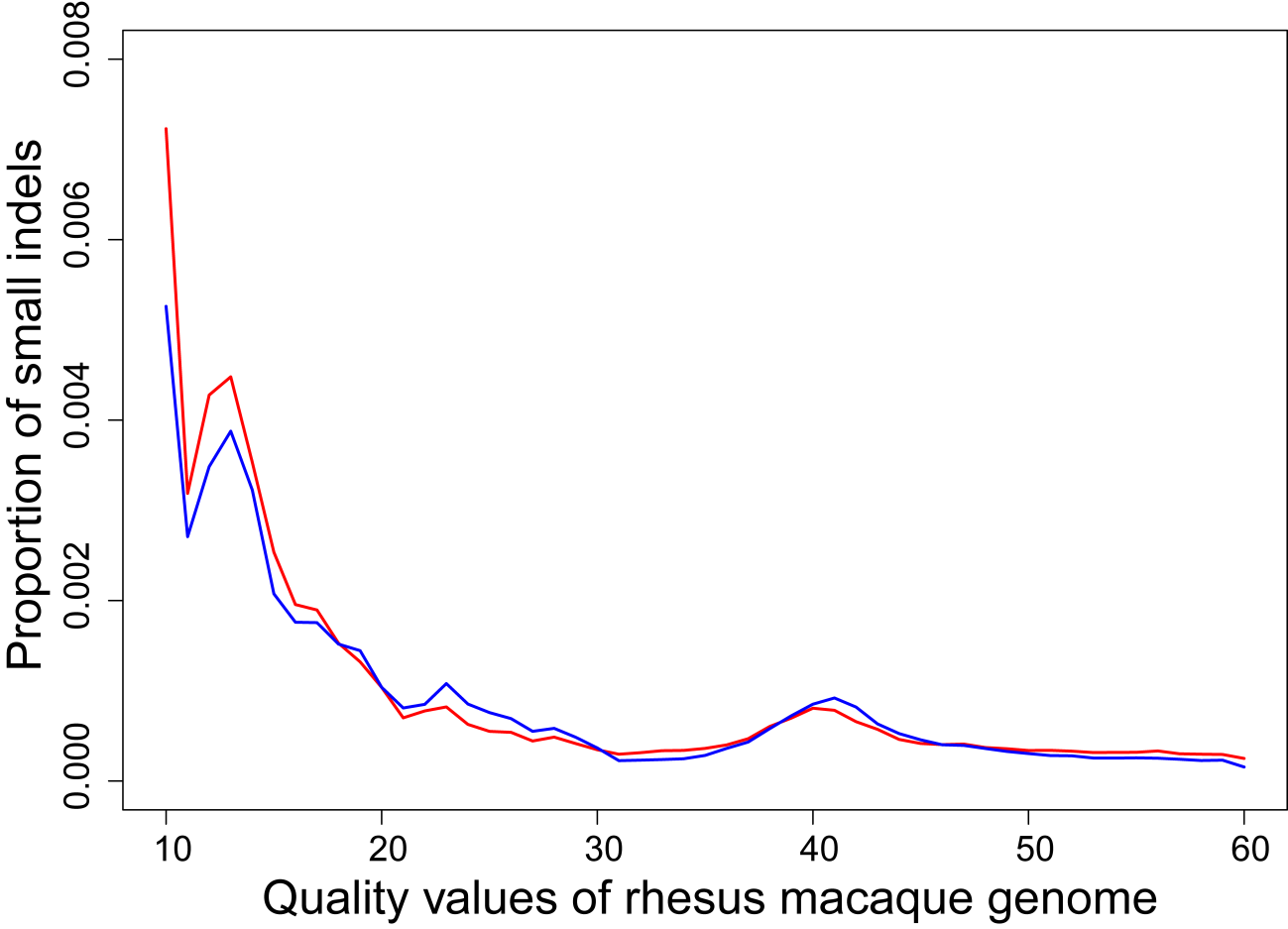


**Supplementary figure 4**

Small indel discovery rate and rhesus macaque genome quality. The red and blue lines represent the rate of small deletions and insertions, respectively, with given rhesus macaque genome sequence quality values (QVs). Small indels at sites having QV <45 in the rhesus macaque genome sequence were filtered out.


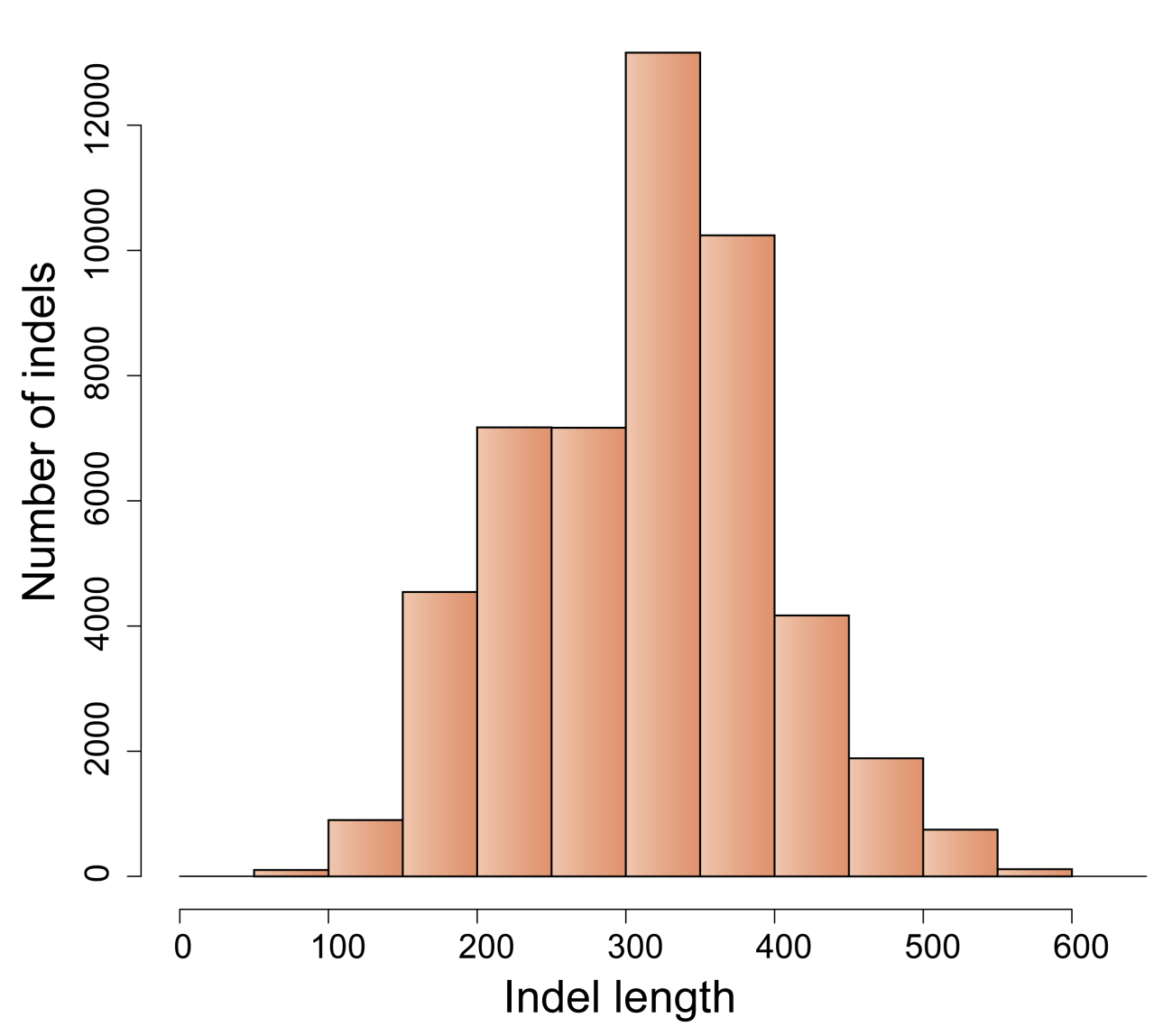


**Supplementary figure 5**

Distribution of large-indel lengths identified in the cynomolgus macaque genome. Indels were identified using the distance information from the mate-pair libraries. Indel regions containing ambiguous genome sequences were excluded.

**Supplementary Table 1. Summary of SOLiD libraries and sequence reads (mapped to the Vietnamese cynomolgus macaque genome sequence)**

| Library | Read length (bp) | Insert size (bp) | Runs | Reads | Mapped reads | Analyzed reads^*^ | Coverage depth of analyzed reads |
| --- | --- | --- | --- | --- | --- | --- | --- |
| Fragment | 50 | - | 8 | 2,648,128,521 | 1,910,075,224  (72.1%) | 1,909,361,950 (72.1%) | 34.8 |
| Mate-pair A | 25 (×2) | 600–800 | 2 | 906,783,481 | 595,034,829  (65.6%) | 309,678,624^**^ (34.2%)^**^ | 3.2 |
| Mate-pair B | 25 (×2) | 800–1,000 | 2 | 1,335,583,547 | 779,082,147  (58.3%) | 439,257,710^**^ (32.9%)^**^ | 4.5 |
| Total | - | - | 12 | 4,890,495,549 | 3,284,192,200 (67.2%) | 2,658,298,284  (54.4%) | 42.5 |

* Reads mapped on chrM and chrUr were removed.

** “PCR or optical duplicates” (defined by Bioscope; mapped more than 100 loci) were removed, and properly paired reads were selected; each read of a pair was mapped on the same chromosome in a proper direction at a proper distance from each other.

**Supplementary Table 2. Pattern of nucleotide changes**

| Substitution type (reference−>variant) | Number |
| --- | --- |
| A−>C | 390,138 |
| A−>G | 1,653,142 |
| A−>T | 249,718 |
| C−>A | 371,595 |
| C−>G | 415,450 |
| C−>T | 1,752,266 |
| G−>A | 1,755,150 |
| G−>C | 416,160 |
| G−>T | 371,953 |
| T−>A | 250,197 |
| T−>C | 1,651,323 |
| T−>G | 390,290 |
| Transition | 6,811,881 |
| Transversion | 2,855,501 |

**Supplementary Table 3. Immune- and drug-response genes with completely segregating nonsynonymous SNVs between cynomolgus and rhesus macaques**

| Gene symbol | NCBI Gene ID | Gene Ontology | Number of fixed non-synonymous changes | Changes | Chromosome |
| --- | --- | --- | --- | --- | --- |
| 11-BETA-HSD2 | 3291 | response to drug | 2 | T65731509C; C65738792T | chr20 |
| LOC713636 | 8412 | response to drug | 2 | T96594747C; A96594856G | chr1 |
| GIP | 2695 | response to drug | 2 | G33191832A; T33191922G | chr16 |
| XRCC1 | 7515 | response to drug | 1 | T50005393G | chr19 |
| TNFRSF11B | 4982 | response to drug | 1 | A121454472G | chr8 |
| ABCB1 | 5243 | response to drug | 1 | C129670032T | chr3 |
| NOS3 | 4846 | response to drug | 1 | G187955536C | chr3 |
| CENPF | 1063 | response to drug | 1 | G155817360T | chr1 |
| HMGCS2 | 3158 | response to drug | 1 | C122682266T | chr1 |
| USP47 | 55031 | response to drug | 1 | A59964128G | chr14 |
| GNAS | 2778 | response to drug | 1 | T5677012G | chr10 |
| MVD | 4597 | response to drug | 1 | G86810862C | chr20 |
| LRP2 | 4036 | response to drug | 1 | G32898560A | chr12 |
| LCT | 3938 | response to drug | 1 | G116340193A | chr13 |
| ATG5 | 9474 | response to drug | 1 | T102253021C | chr4 |
| ABCG5 | 64240 | response to drug | 1 | C43959921T | chr13 |
| MAP1B | 4131 | response to drug | 1 | A68345766G | chr6 |
| LTC4S | 4056 | response to drug | 1 | G176476568A | chr6 |
| LOC700588 | 8744 | immune response | 3 | T6438112C; G6438272C; G6441336A | chr19 |
| CIITA | 4261 | immune response | 2 | A10975203G; G10978514A | chr20 |
| MAP3K14 | 9020 | immune response | 2 | A55363185G; A55364227G | chr16 |
| IL1RL1 | 9173 | immune response | 2 | T102264989G; C102271985T | chr13 |
| LOC714202 | 1380 | immune response | 2 | T162884203G; T162887990C | chr1 |
| TNFRSF1B | 7133 | immune response | 2 | C15321283T; G15321403A | chr1 |
| TLR4 | 7099 | immune response | 2 | T18365726G; C18366037T | chr15 |
| LOC710234 | 6932 | immune response | 2 | A130552066G; T130552112C | chr6 |
| LOC713238 | 116449 | immune response | 2 | A5462457G; T5462493G | chr5 |
| CXCL13 | 10563 | immune response | 1 | G51961099C | chr5 |
| CCL11 | 6356 | immune response | 1 | A29581970G | chr16 |
| CD276 | 80381 | immune response | 1 | A52386535G | chr7 |
| SAMHD1 | 25939 | immune response | 1 | T27505202C | chr10 |
| IGSF6 | 10261 | immune response | 1 | A20920779C | chr20 |
| IL2RG | 3561 | immune response | 1 | C70018992T | chrX |
| LOC712754 | 5996 | immune response | 1 | A178029785G | chr1 |
| LOC714474 | 7454 | immune response | 1 | C46544519T | chrX |
| FCAR | 2204 | immune response | 1 | A60896070G | chr19 |
| LOC696887 | 3550 | immune response | 1 | C137066546G | chr6 |
| CHIT1 | 1118 | immune response | 1 | T167211141C | chr1 |
| VPREB1 | 7441 | immune response | 1 | A65787236G | chr10 |
| IL1F8 | 27177 | immune response | 1 | A112399065G | chr13 |
| TGFBR3 | 7049 | immune response | 1 | T94714589C | chr1 |
| TLR6 | 10333 | immune response | 1 | C33810360T | chr5 |
| CCL27 | 10850 | immune response | 1 | A42861623G | chr15 |
| HFE | 3077 | immune response | 1 | T26087275C | chr4 |
| CD274 | 29126 | immune response | 1 | T71696647A | chr15 |
| VAV1 | 7409 | immune response | 1 | A6745246G | chr19 |
| CTSC | 1075 | immune response | 1 | A86496817C | chr14 |
